# Prevalence, risk factors, and prognosis of central nervous system manifestations in antiphospholipid syndrome

**DOI:** 10.1038/s41598-023-35955-2

**Published:** 2023-06-01

**Authors:** Meige Liu, Gongming Li, Xiaodong Song, Yangyi Fan, Chun Li

**Affiliations:** 1 Department of Neurology, Peking University People’s Hospital, No.11 Xizhimen South Street, Xicheng District, Beijing, 100044 China; 2 Department of Rheumatology and Immunology, Linyi Traditional Chinese Medicine Hospital, Shandong, China; 3 Department of Rheumatology and Immunology, Peking University People’s Hospital, No.11 Xizhimen South Street, Xicheng District, Beijing, 100044 China

**Keywords:** Medical research, Neurology, Rheumatology, Risk factors

## Abstract

The central nervous system (CNS) is considered as one of the most frequently affected organs in antiphospholipid syndrome (APS). This study investigated the prevalence of CNS manifestations in APS and associated risk factors and evaluated stroke recurrence. We carried out this retrospective study from 2009 to 2021 at Peking University People’s Hospital, which enrolled 342 APS patients, and 174 neurologic events were suffered by 119 patients (34.8%). Patients with and without CNS involvement were compared regarding demographics and laboratory parameters. The analysis showed that older age, livedo reticularis, and dyslipidaemia were significant related factors for CNS manifestations (*P* = 0.047, 0.038, and 0.030 respectively). The use of anticoagulants (*P* = 0.004), and/or hydroxychloroquine (*P* = 0.016) appeared to associated with a lower incidence of CNS manifestations. During a median follow-up of 4.1 years, 10 individuals developed new episodes of stroke in APS patients with previous ischemic strokes. Livedo reticularis, smoking and male gender may predict the risk of recurrent stroke (*P* = 0.020, 0.006, and 0.026 respectively). Collectively, our results indicated the protective and risk factors for CNS manifestations, as well as demonstrated that APS patients appeared at high risk of stroke recurrence despite current therapy.

## Introduction

As an autoimmune pro-thrombotic condition, antiphospholipid syndrome (APS) is featured by thrombosis (arterial, venous, or both), obstetric morbidity, and the presence of persistent antiphospholipid antibodies (aPLs)^1^. Three types of antibodies, such as anti-β2 glycoprotein I (aβ2GPIs) and anticardiolipin (aCL) antibodies, as well as the lupus anticoagulant (LA), are contained in the aPLs^2^. In individuals who are less than 50 years old, 20–30% of strokes are considered as APS^3^.

In APS, the CNS is considered the most frequently affected organs. The transient ischemic attack (TIA) and stroke are defined as the thrombotic events frequently observed during follow up, and most of the death in the Euro-Phospholipid APS patients is caused by stroke^4,5^. The prevalence of each clinical phenotype of CNS manifestations in patients with APS varies from different areas of the world. The prevalence of stroke ranges from 19.8 to 38.6%^6–8^. The disparities might be due to differences in ethnicities, environment, and age.

For ischemic stroke, the age, gender, and the risk factors of cardiovascular, such as smoking habits, hyperlipidaemia, and hypertension, were considered as the risk factors^9–11^. In addition, age was shown to be associated with cognitive dysfunction and psychiatric symptoms in APS^12^. While, because of the used laboratory assays, study populations’ variation, and relatively small sample sizes, the interpretability is limited in previous studies^9–12^. Additionally, in the APS patients from China, the risk factors for the manifestations of overall CNS are rarely investigated. As CNS manifestations represent one of the most severe complications and are responsible for significant morbidity and mortality^4–6^, therefore, clarifying the potential risk factors, identifying the markers for prognosis, and discovering the measures for therapy are urgently needed to prevent the APS patients from these important complications. Herein, we analyzed the risk profiles, clinical features, and prevalence for the manifestations of CNS in a cohort of APS patients from China, and evaluated the rate and predictive factors of recurrent stroke during follow-up, which may provide a basis for effective prevention and therapy strategies.

## Methods

### Patients

The Peking University People’s Hospital Ethics Committee approved this observational retrospective cohort study (2019PHB252) and the waiver of Informed Consent. The study was carried out according to the principles of the Declaration of Helsinki.

We consecutively collected data from definitive APS patients at Peking University People’s Hospital from January 2009 through December 2021. The inclusion criteria were: (1) patients fulfilled the 2006 Sydney classification criteria for APS^1^ and were confirmed by at least two expert rheumatologists, (2) age ≥ 18 years, (3) available for long-term follow-up. The exclusion criteria were: (1) patients who had CNS manifestations caused by any other known disorder, such as trauma, brain tumor, and corticosteroid-induced psychotic disorder, (2) severe liver disease, malignancy, coagulopathy, and various chronic or acute infections, (3) incomplete clinical medical record data.

### Clinical and demographic data

The patients’ medical charts were given for retrospective and exhaustive review, which included detailed demographic, clinical, and laboratory information. Variables collected included the following: age, gender, disease duration, obstetric complications, extracranial sites of thrombosis (arterial or venous), CNS manifestations, non-criteria manifestations of APS (livedo reticularis, cardiac valve disease, diffuse alveolar haemorrhage, APS nephropathy, thrombocytopenia [platelet counts < 100 × 10^9^/L]), hypertension, diabetes mellitus, dyslipidaemia, smoking, and obesity (body mass index [BMI] > 30 kg/m^2^). Treatments with antiplatelet (aspirin), anticoagulants (warfarin, new direct oral anticoagulant or low-molecular weight heparin), corticosteroids, hydroxychloroquine (HCQ), and immunosuppressants were also recorded.

CNS manifestations of APS were diagnosed by the experienced neurologist, psychiatrist, and ophthalmologist using reliable objective examination results^3^. Cerebrovascular disease, such as ischemic stroke, TIA, and cerebral venous thrombosis, were considered as the acute onset of focal neurologic deficit and confirmed with radiological evidence. The diagnosis of cognitive deficit was based on International Classification of Diseases, 10th revision (ICD-10) diagnostic code^13^. Migraine was defined according to the International Migraine Society diagnostic criteria^14^. Ocular syndrome was defined as amaurosis fugax and optic neuropathy^15^. The classification of psychiatric disorders were defined according to the ICD-10 criteria, including depression, anxiety, delusion, bipolar disorders, and hallucination^13^. The disorders of movement included chorea dyskinesia, parkinsonism, and cerebellar ataxia.

### Detection of aPLs

The Stago STA Compact Hemostasis System was applied to complete the LA assay, patients were considered positive for LA if the simplified Dilute Russell’s Viper Venom Test (dRVVT) ratios (LA1 screen/LA2 confirmation) were > 1.2. The levels of aβ2GP1 antibody (EUROIMMUN Medical Laboratory Diagnostics, Germany) and aCL (AESKU Group, Wendelsheim, Germany) were measured by ELISA assay. Based on local cutoff, values for aβ2GP1 > 27 RU/mL and aCL > 12 IU/mL were classified as positive^16^.

### Follow up

We started the follow-up when the diagnosis involved in CNS was performed in APS patients, and then performed no less than 6 months (telephone contact or visit at center). The observation index was recurrent infarcts of brain in APS patients with a known ischemic stroke. Additionally, according to the clinical criteria based on typical clinical syndrome (a new and acute onset of focal neurological deficit lasting > 24 h) with radiological evidence of brain infarction, a neurologist was employed to identify the recurrent stroke. If a recurrence of stroke was observed, the follow-up will be terminated. The status of recurrence was examined on February 5th, 2022. If no death or recurrence was observed, the date for examination was used as the last day of follow-up.

### Statistical analysis

The SPSS software was applied to analyze the collected data in this study. The continuous variables of normal and non-normal distribution were presented as mean ± standard deviation (SD) and median and interquartile range, respectively. The categorical variables were presented as numbers and percentages. Patients with and without CNS involvement were compared via statistical analyses regarding demographics and laboratory parameters. The Mann Whitney-U test and Student’s t-test were carried out to evaluate the differences of indicated study groups. The chi-squared test was applied to access the difference of the categorical data. Variables, such as age, gender, secondary APS, aPLs, treatment at disease onset, and the risk factors of traditional cardiovascular including smoking, dyslipidaemia, hypertension, and diabetes mellitus, which may reasonably be associated with CNS manifestations were investigated using the models of univariate logistic regression. If the p-value of these factors obtained from the univariate analysis less than 0.05, and the multiple logistic regression models will be applied to determine the 95% confidence intervals (95% CIs) and odds ratios (ORs).

For evaluating the predictors of recurrent stroke, we carried out the univariate Cox proportional hazard analysis. The interval of the stroke first recurrence and the ischemic stroke first diagnosis in APS patient was set as the time to recurrence. The significant difference was considered as the p-value of a 2-sided probability less than 0.05.

## Results

### Clinical characteristics of the APS group

**Figure 1:**
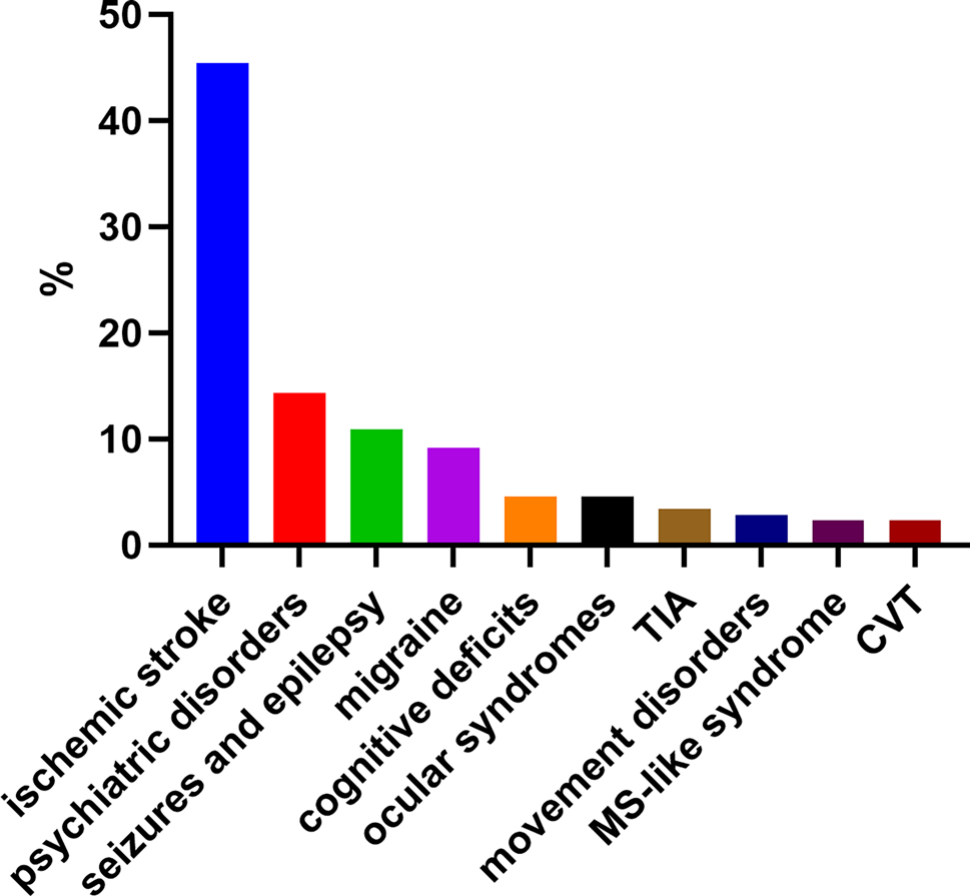
Types and frequencies of CNS manifestations in patients with APS. *CNS* central nervous system, *APS* antiphospholipid syndrome, *TIA* transient ischemic attack, *MS* multiple sclerosis, *CVT* cerebral venous thrombosis.

The population of the study consisted of 342 patients (266 female and 76 male), aged 44.1 ± 16.3 years (range 16–88 years; Table 1). While 51.8% (177/342) of the patients had primary APS, in 48.2% (165/342) APS was secondary and associated with autoimmune diseases, 126 with systemic lupus erythematosus (Supplementary Table 1), 39 patients with other autoimmune disorders (18 with Sjögren’s syndrome, 14 with rheumatoid arthritis, and 7 with systemic sclerosis) (Supplementary Table 2). Among the patients, 34.8% (119/342) developed 174 neurologic events (Fig. 1). For those who had CNS manifestations, the average number of events per patient was 1.46 (range 1–4). In detail, 70.6% (84/119) of the patients with CNS manifestations experienced one neurological symptom, while 20.2% (24/119), 5.9% (7/119), and 3.4% (4/119) experienced 2, 3, and 4 concurrent CNS manifestations, respectively. Ischemic stroke was the most frequent CNS manifestations recorded (79/174, 45.4%) and followed by psychiatric manifestations (25/174, 14.4%), seizures and epilepsy (19/174, 10.9%), migraine (16/174, 9.2%), cognitive deficits (8/174, 4.6%), ocular syndromes (8/174, 4.6%), TIA(6/174, 3.4%), movement disorders (5/174, 2.9%), multiple sclerosis-like disease (4/174, 2.3%), and cerebral venous thrombosis (4/174, 2.3%).

**Table 1:** General characteristics of the study population.

Variable	
Male gender, n (%)	76 (22.2)
Age at diagnosis (years)	44.1 ± 16.3
Secondary APS, n (%)	165 (48.2)
APS duration (months)	8 (1, 36)
Isolated thrombotic APS, n (%)	214 (62.6)
Isolated obstetric APS, n (%)	98 (28.7)
Thrombotic + obstetric APS, n (%)	30 (8.8)
Arterial thrombosis, n (%)	169 (49.4)
Ischemic stroke, n (%)	79 (23.1)
Pulmonary embolism, n (%)	48 (14.0)
Myocardial infarction, n (%)	30 (8.8)
Renal arterial thrombosis, n (%)	8 (2.3)
Aortic thrombosis, n (%)	6 (1.8)
Limb artery thrombosis, n (%)	15 (4.4)
Retinal artery thrombosis, n (%)	2 (0.6)
Venous thrombosis, n (%)	134 (39.2)
Cerebral venous thrombosis, n (%)	4 (1.2)
Renal venous thrombosis, n (%)	3 (0.9)
Superior/inferior vena cava thrombosis, n (%)	11 (3.2)
Limb venous thrombosis, n (%)	114 (33.3)
Retinal vein thrombosis, n (%)	3 (0.9)
CNS involvement, n (%)	119 (34.8)
≥ 1 cardiovascular risk factor, n (%)	256 (74.9)

Categorical variables are described as n (%), and continuous variables as mean ± standard deviation, or median (interquartile range).

*APS* antiphospholipid syndrome, *CNS* central nervous system.

### Distribution of risk factors, associated comorbidities, and treatment

In comparison to the patients without CNS manifestations, patients with CNS manifestations had longer disease duration (median 10 [interquartile range (1, 48)] *vs.* 7 [interquartile range (1, 36)] months, *P* = 0.033), were older (49.7 ± 16.1 *vs.* 41.2 ± 15.7 years, *P* < 0.001), and had a higher rate of secondary APS (63.0% *vs.* 40.0%, *P* < 0.001; Table 2). Regarding the clinical manifestations, significantly high rates of extracranial arterial events (43.7% *vs.* 32.3%, *P* = 0.037), livedo reticularis (7.6% *vs.* 2.2%, *P* = 0.023), and thrombocytopenia (38.7% *vs.* 26.0%, *P* = 0.015) were observed in the group with CNS manifestations in comparison to the group without CNS manifestations. In addition, patients with CNS manifestations were significantly more likely to experience the traditional cardiovascular risk factors, such as dyslipidaemia (74.8% *vs.* 12.9%, *P* < 0.001), hypertension (45.4% *vs.* 22.7%, *P* < 0.001), and diabetes mellitus (22.7% *vs.* 8.9%, *P* < 0.001). Compared with the group without CNS manifestations, the group with CNS manifestations showed significantly higher rate of LA antibody positivity (73.9% *vs.* 62.7%, *P* = 0.045), whereas the differences in aCL (75.6% *vs.* 66.2%, *P* = 0.091) and triple positivity (38.7% *vs.* 34.1%, *P* = 0.400) were less significant.

**Table 2:** Demographic and clinical features of APS patients with and without CNS manifestations.

	With CNS manifestations (n = 119)	Without CNS manifestations (n = 223)	*P *
Age at diagnosis (years)	49.7 ± 16.1	41.2 ± 15.7	< 0.001
Male gender, n (%)	33 (27.7)	43 (19.1)	0.073
Disease duration (months)	10 (1, 48)	7 (1, 36)	0.033
Secondary APS, n (%)	75 (63.0)	90 (40.0)	< 0.001
Extracranial arterial events, n (%)	52 (43.7)	72 (32.3)	0.037
Venous events, n (%)	49 (41.2)	85 (38.1)	0.638
Obstetric events, n (%)	25 (21.0)	104 (46.2)	< 0.001
Livedo reticularis, n (%)	9 (7.6)	5 (2.2)	0.023
Diffuse alveolar hemorrhage, n (%)	1 (0.8)	0 (0)	1.000
APS nephropathy, n (%)	3 (2.5)	2 (0.9)	0.347
Thrombocytopenia, n (%)	46 (38.7)	58 (26.0)	0.015
Diabetes, n (%)	27 (22.7)	20 (8.9)	< 0.001
Hypertension, n (%)	54 (45.4)	51 (22.7)	< 0.001
Dyslipidaemia, n (%)	89 (74.8)	29 (12.9)	< 0.001
Smoking, n (%)	22 (18.5)	30 (13.3)	0.217
Obesity, n (%)	10 (8.4)	23 (10.3)	0.569
aβ2GPI, n (%)	75 (63)	161 (71.6)	0.081
aCL, n (%)	90 (75.6)	149 (66.2)	0.091
LA, n (%)	88 (73.9)	141 (62.7)	0.045
Triple aPL positivity, n (%)	46 (38.7)	76 (34.1)	0.400
Antiplatelet drugs, n (%)	46 (38.7)	82 (36.4)	0.732
Anticoagulants, n (%)	53 (44.5)	148 (65.8)	< 0.001
Corticosteroids, n (%)	99 (83.2)	155 (68.9)	0.006
Hydroxychloroquine, n (%)	60 (50.4)	167 (74.2)	< 0.001
Immunosuppressants, n (%)	102 (85.7)	193 (86.5)	0.831
Statins, n (%)	15 (12.6)	131 (58.2)	< 0.001

Data were presented as the mean ± standard deviation, median (interquartile range) or n, where n = number of patients.

*APS* antiphospholipid syndrome, *CNS* central nervous system, *aβ2GPIs* anti-β2 glycoprotein I antibodies, *aCL* anticardiolipin antibody, *LA* lupus anticoagulant, *aPL* antiphospholipid antibody.

The treatment regimens of the two groups that were administered at disease onset were compared. Patients in the group without CNS manifestations were significantly more likely to receive anticoagulants (65.8% *vs.* 44.5%, *P* < 0.001), HCQ (74.2% *vs.* 50.4%, *P* < 0.001), or statins (58.2% *vs.* 12.6%, *P* < 0.001). Patients without CNS manifestations received corticosteroids at a lower rate than those patients with CNS manifestations (68.9% *vs.* 83.2%, *P* = 0.006). The usage of antiplatelet drug (38.7% *vs.* 36.4%, *P* = 0.732) and immunosuppressants (85.7% *vs.* 86.5%, *P* = 0.831) of the 2 groups were comparable.

The analysis of multivariate logistic regression demonstrated that the patients with CNS manifestations were older (OR: 1.017, 95% CI: 1.000–1.034, *P* = 0.047), and significantly more likely to experience livedo reticularis (OR: 3.833, 95% CI: 1.075–13.669, *P* = 0.038), and dyslipidaemia (OR: 1.903, 95% CI: 1.065–3.402, *P* = 0.030), compared with the patients without CNS manifestations (Table 3). Treatment with anticoagulants (OR: 0.446, 95% CI: 0.256–0.775, P = 0.004), and HCQ (OR: 0.509, 95% CI: 0.294–0.882, *P* = 0.016) appeared to decrease the incidence of CNS manifestations.

**Table 3:** Univariate and multivariate logistic regression analyses of factors associated with CNS manifestations.

	Univariate analysis			Multivariate analysis		
	OR	95% CI	*P*	OR	95% CI	*P*
Age at diagnosis	1.032	1.017, 1.046	< 0.001	1.017	1.000, 1.034	0.047
Male gender	1.605	0.953, 2.702	0.075	0.769	0.404, 1.463	0.423
Disease duration	1.004	1.000, 1.008	0.033	1.004	1.000, 1.009	0.075
Secondary APS	2.581	1.628, 4.093	< 0.001	1.467	0.821, 2.621	0.195
Extracranial arterial events	10.306	5.905, 17.987	0.002	1.305	0.740, 2.302	0.358
Venous events	1.124	0.713, 1.774	0.581			
Obstetric events	0.300	0.179, 0.505	< 0.001	0.532	0.265, 1.070	0.077
Livedo reticularis	3.667	1.200, 11.206	0.026	3.833	1.075, 13.669	0.038
APS nephropathy	2.858	0.471, 17.345	0.254			
Thrombocytopenia	1.793	1.115,2.883	0.016	1.052	0.589,1.880	0.864
Diabetes	2.979	1.589, 5.585	0.001	2.103	0.991, 4.462	0.053
Hypertension	2.924	1.811, 4.722	< 0.001	1.534	0.865, 2.720	0.143
Dyslipidaemia	2.436	1.383, 4.292	< 0.001	1.903	1.065, 3.402	0.030
Smoking	1.505	0.824, 2.749	0.219			
Obesity, BMI > 30	1.253	0.576, 2.730	0.569			
aβ2GPI	0.684	0.426, 1.099	0.098			
aCL	1.548	0.936, 2.558	0.065			
LA	1.728	1.053, 2.835	0.038	1.286	0.685, 2.415	0.434
Triple aPL positivity	1.219	0.768, 1.933	0.400			
Antiplatelet drugs	1.130	0.713, 1.789	0.732			
Anticoagulants	0.416	0.264, 0.657	< 0.001	0.446	0.256, 0.775	0.004
Corticosteroids	2.190	1.254, 3.827	0.004	1.562	0.744, 3.278	0.239
Hydroxychloroquine	0.353	0.221, 0.565	< 0.001	0.509	0.294, 0.882	0.016
Immunosuppressants	0.933	0.491, 1.771	0.831			
Statins	2.398	1.100, 5.228	0.033	1.503	0.615, 3.673	0.371

*CNS* central nervous system, *APS* antiphospholipid syndrome, *95% CI* 95% confidence interval, *BMI* body mass index, *aβ2GPIs* anti-β2 glycoprotein I antibodies, *aCL* anticardiolipin antibody, *LA* lupus anticoagulant, *aPL* antiphospholipid antibody.

### Recurrent stroke

During a median follow-up of 4.1 years (range 0.5–15 years), 12.7% patients (10/79) still developed recurrent infarcts of brain in APS patients with a known ischemic stroke despite current treatment (Supplementary Table 3). Recurrent stroke was predicted by livedo reticularis (HR: 6.296, 95% CI: 1.335–29.684, *P* = 0.020), smoking (HR: 5.697, 95% CI: 1.638–19.812, *P* = 0.006) and male gender (HR: 4.120, 95% CI: 1.180–14.389, *P* = 0.026) (Table 4).

**Table 4:** Univariate Cox regression analyses of predictors of ischemic stroke recurrence in APS patients.

	Univariate analysis		
	HR	95% CI	*P*
Age at diagnosis	1.018	0.981, 1.056	0.345
Male gender	4.120	1.180, 14.389	0.026
Disease duration	0.999	0.990, 1.008	0.862
Secondary APS	1.817	0.465, 7.092	0.390
Extracranial arterial events	3.034	0.855, 10.770	0.086
Venous events	1.376	0.397, 4.763	0.615
Obstetric events	0.788	0.203, 3.054	0.731
Livedo reticularis	6.296	1.335, 29.684	0.020
Thrombocytopenia	0.253	0.032, 2.004	0.193
Diabetes	2.308	0.486, 10.959	0.293
Hypertension	2.277	0.659, 7.869	0.193
Dyslipidaemia	5.544	0.702, 43.768	0.104
Smoking	5.697	1.638, 19.812	0.006
Obesity, BMI > 30	1.079	0.137, 8.519	0.943
aβ2GPI	4.109	0.521, 34.442	0.180
aCL	0.523	0.132, 2.075	0.357
LA	3.778	0.478, 29.880	0.208
Triple aPL positivity	1.804	0.505, 6.448	0.364
Antiplatelet drugs	2.455	0.693, 8.703	0.164
Anticoagulants	0.849	0.245, 2.941	0.796
Corticosteroids	1.325	0.280, 6.260	0.722
Hydroxychloroquine	1.024	0.287, 3.659	0.970
Immunosuppressants	1.229	0.155, 9.724	0.845
Statins	3.659	0.756, 17.717	0.107

*APS* antiphospholipid syndrome, *95% CI* 95% confidence interval, *BMI* body mass index, *aβ2GPIs* anti-β2 glycoprotein I antibodies, *aCL* anticardiolipin antibody, *LA* lupus anticoagulant, *aPL* antiphospholipid antibody.

## Discussion

Our study found that 34.8% of the overall population experienced CNS manifestations, which was similar to the rate reported in a study from Israel (35.4%)^17^. The multivariate analysis revealed that older age, livedo reticularis, and dyslipidaemia were independent risk factors of CNS manifestations, while anticoagulants, HCQ, or a combination of both may be protective against CNS manifestations. Patients with CNS manifestations of ischemic stroke still showed a high rate of recurrent stroke despite current therapy, livedo reticularis, smoking and male gender may be predictors of ischemic stroke recurrence.

A positive connection of the cardiovascular risk factors presence to the increased thrombotic risk was discovered in APS patients. In a retrospective study of 138 aPL carriers, the smoking and hypertension were considered as the risk factors for thrombosis development independently^18^. Additionally, the associations of the cerebrovascular events with high-titre aCL, with hyperlipidaemia and smoking were reported^19^. In line with these results, the associations of the recurrent events with the presence of cigarette smoking and hyperlipidaemia were also observed in a study for neurological and cerebrovascular disease and aPLs using 48 cases^20^. Consistent with previous reports, the present study found that dyslipidaemia was significantly higher in those with CNS manifestations, and smoking might be an independent predictor for recurrent ischemic stroke. This supports that dyslipidaemia should be treated properly and smoking cessation is recommended to minimize the risk of CNS involvements in APS.

Livedo reticularis is a non-criteria manifestation of APS and the most common cutaneous symptom, resulting from a narrowing of the medium and small arteries at the dermis-subcutis border^21–23^. Herein, we found that the CNS manifestations were more likely observed in APS patients if they had livedo reticularis, which was consisted with the previous studies^11,23–25^, and recurrent stroke might be predicted by livedo reticularis. The present findings may suggest that livedo reticularis may be a sign of higher risk for CNS manifestations in APS and require a closer follow-up. Previous studies have found that the small- and medium-size cerebral arteries-involved noninflammatory thrombotic vasculopathy comparable to the pathology noted in dermal arteries^26,27^. The speculation that changes in both skin and CNS reflecting the same thrombotic processes was significantly supported by the connection of CNS manifestations to the livedo reticularis presence. But the specific mechanism is still unknown. One hypothesis to explain the finding is that both of brain and skin have same ectodermal origin^28^. And another one is the systemic arterial bed dysfunctions in APS, such as arterial beds in the skin, lungs, kidneys, heart and brain^29^. These hypotheses have yet to be explored and further studies are necessary.

For the treatment of CNS manifestations in APS patients, the supportive and definitive data are limited. In APS patients who present with thrombotic events and cerebral ischemia, the long-term anticoagulation administration with warfarin is considered as the mainstay treatment strategy^30,31^, but for the APS with non-thrombotic CNS manifestations, the evidences about the options of specific treatment were mostly from small-scale reports. Several studies reported that non-thrombotic CNS manifestations, such as migraine and memory loss, were improved by anticoagulation for other APS manifestations. The worsening headaches were reported by many patients when the international normalized ratio (INR) became subtherapeutic^32,33^. Additionally, after 2- to 4-week treatment with anticoagulation, clopidogrel, or aspirin, 94%, 83%, and 47% of the patients with aPL and refractory migraine had a symptomatic response, respectively^34^. Moreover, a major clinical response, like an improvement in severity or frequency of migraine, or both, were observed in the 85% patients from anticoagulated group, and no patient experienced major bleeding^34^. In the present study, administration of anticoagulants was tightly connected to the lower rate of CNS manifestations. Therefore, long-term usage of anticoagulation for the APS treatment was confirmed by our present data, although the optimal treatment should be determined by specific randomized trials in further.

The protection of HCQ on APS patients against thrombosis was also reported by in vitro and in vivo studies^35–39^. Recently, using a pilot open-label randomized prospective study, the efficacy and safety of HCQ for the prevention of thrombosis was examined in 50 primary APS patients^40^. It was found that HCQ combined with standard treatment was significantly connected to a reduction in aPLs titres over an average 2.6-year follow-up and a lower rate of thrombosis in comparison to the standard treatment alone. In the present cohort, we observed a significant connection of the HCQ usage to the lower risk of CNS manifestations in these APS patients, suggesting its potential role in the prevention of CNS manifestations in APS. However, the link between them should be further verified by more studies. Some published studies believed that the protective function of HCQ is closely connected to the reduction of the aPL titre^39,41^, and its pleiotropic antithrombotic, metabolic, immunoregulatory, and anti-inflammatory effects^42,43^. These observations should be confirmed by appropriately designed population studies.

However, no significant correlation of aPLs (aβ2GP1, aCL, LA) to CNS manifestations in APS was found. This was consistent with Volkov et al.^17^, in which no criteria aPLs (aβ2GP1, aCL) were associated with APS-related CNS manifestations in 130 patients. These results demonstrated that the adverse clinical outcomes cannot be caused by the aPLs alone, and additional risk factors, especially cardiovascular risk factors, might accelerate clinical manifestations in patients with persistent aPLs. These results further confirmed the “second hit” model of thrombus formation in APS^44^. The aPLs presence is considered as the “first hit” because of the promotion of a procoagulant state, while the thrombotic event is connected to the occurrence of the “second hit”. Therefore, strict control and regular assessment of modifiable cardiovascular risk factors is of vital importance to be recommended for all aPLs-positive individuals, such as hypertension management, smoking cessation, dyslipidaemia, and diabetes^16,45^.

In APS, the risk factors closely connected to the recurrence of stroke have not completely uncovered yet. If aPLs closely connected to the recurrence of stroke in patients with APS is not confirmed^46^. As for cardiovascular risk factors, previous studies showed that cigarette smoking, hyperlipidaemia, and arterial hypertension were significantly associated with recurrent cerebrovascular events^20,47^. In our cohort, the rate of stroke recurrence was 12.7%, which was higher than the Euro-Phospholipid cohort^48^. This may be attributed to older age, poor adherence to treatment (withdrawal or poor control of anticoagulation intensity), and high prevalence of cardiovascular risk factors. Our results showed that livedo reticularis, smoking and male gender were more common in the recurrent stroke group than those in the patients without stroke recurrence. The findings may further support the notion that a rigid control of smoking and aggressive treatment of livedo reticularis in APS. However, considering the small number of recurrent stroke in our study, we only used univariate Cox proportional hazard analysis to evaluate the predictors of recurrent stroke, and the multivariate Cox proportional hazard analysis was not performed. Therefore, there might result in weak associations and it is difficult to get a conclusion about the definite predictors for stroke recurrence in APS. More researches with larger samples are needed for further investigation, for these patients might be in the highest risk of recurrent thrombosis and finally functional disability.

However, several limitations are also existed. First, we can’t obtain any data from medical reports that was not originally recorded, and for each patient’s follow-up time, a marked variability is also existed. Consequently, the findings may be vulnerable to information bias. Second, APS incidence is relative low^3^, and the sample size collected from a single centre was relatively small, and patients with APS from diverse ethnic and genetic backgrounds were not included. Therefore, the current results may not be generalizable. Third, the type of anticoagulants varied among patients, and anticoagulation monitoring was not systematically evaluated during the follow-up. It is hard to make sure what would be the optimal INR target for anticoagulation treatment and to determine whether a particular anticoagulant was more favourable than others. Further large-scale, prospective, randomized clinical studies are warranted.

## Conclusions

The potential risk factors for CNS manifestations among patients with APS include older age, dyslipidaemia and livedo reticularis. Moreover, livedo reticularis, smoking and male gender may predict recurrent stroke risk. The risk of CNS manifestations in APS may be minimized by controlling modifiable risk factors of cardiovascular, like dyslipidaemia and smoking. Additionally, anticoagulants and HCQ may be effective and relatively safe protective treatments against CNS manifestations in individuals with APS.

## Supplementary Information

Supplementary Tables.

## Data Availability

Please contact the corresponding author for the request of the data used in current study.

## References

[1] Miyakis S (2006). International consensus statement on an update of the classification criteria for definite antiphospholipid syndrome (APS). J. Thromb. Haemost..

[2] Shoenfeld Y, Twig G, Katz U, Sherer Y (2008). Autoantibody explosion in antiphospholipid syndrome. J. Autoimmun..

[3] Leal-Rato M, Bandeira M, Romão VC, Aguiar-de-Sousa D (2021). Neurologic manifestations of the antiphospholipid syndrome—an update. Curr. Neurol. Neurosci. Rep..

[4] Cervera R (2014). Morbidity and mortality in the antiphospholipid syndrome during a 10-year period: A multicentre prospective study of 1000 patients. Ann. Rheum. Dis..

[5] Cervera R (2009). Morbidity and mortality in the antiphospholipid syndrome during a 5-year period: A multicentre prospective study of 1000 patients. Ann. Rheum. Dis..

[6] Cervera R (2002). Antiphospholipid syndrome: Clinical and immunologic manifestations and patterns of disease expression in a cohort of 1,000 patients. Arthrit. Rheum..

[7] Shi H (2017). Clinical characteristics and laboratory findings of 252 Chinese patients with anti-phospholipid syndrome: Comparison with Euro-Phospholipid cohort. Clin. Rheumatol..

[8] Grimaud F (2019). Clinical and immunological features of antiphospholipid syndrome in the elderly: A retrospective national multicentre study. Rheumatol. (Oxf.).

[9] Radin M (2018). The risk of ischaemic stroke in primary antiphospholipid syndrome patients: A prospective study. Eur. J. Neurol..

[10] de Souza AW (2007). Impact of hypertension and hyperhomocysteinemia on arterial thrombosis in primary antiphospholipid syndrome. Lupus.

[11] Stojanovich L, Djokovic A, Stanisavljevic N, Zdravkovic M (2018). The cutaneous manifestations are significantly related to cerebrovascular in a Serbian cohort of patients with Hughes syndrome. Lupus.

[12] Chapman J (2002). Prevalence and clinical features of dementia associated with the antiphospholipid syndrome and circulating anticoagulants. J. Neurol. Sci..

[13] Faiad Y (2018). Frequency of use of the International Classification of Diseases ICD-10 diagnostic categories for mental and behavioural disorders across world regions. Epidemiol. Psychiatr. Sci..

[14] Olesen J (2018). International classification of headache disorders. Lancet Neurol..

[15] Ricarte IF (2018). Neurologic manifestations of antiphospholipid syndrome. Lupus.

[16] Li C (2022). Additional risk factors associated with thrombosis and pregnancy morbidity in a unique cohort of antiphospholipid antibody-positive patients. Chin. Med. J. (Engl.).

[17] Volkov I (2020). Profiles of criteria and non-criteria anti-phospholipid autoantibodies are associated with clinical phenotypes of the antiphospholipid syndrome. Auto Immun. Highlight..

[18] Pablo RD (2022). Risk factors for the development of the disease in antiphospholipid antibodies carriers: A long-term follow-up study. Clin. Rev. Allergy Immunol..

[19] Verro P, Levine SR, Tietjen GE (1998). Cerebrovascular ischemic events with high positive anticardiolipin antibodies. Stroke.

[20] Levine SR, Deegan MJ, Futrell N, Welch KM (1990). Cerebrovascular and neurologic disease associated with antiphospholipid antibodies: 48 cases. Neurology.

[21] Gomez-Flores M (2021). Cutaneous manifestations of antiphospholipid syndrome. Lupus.

[22] Petri M (2000). Epidemiology of the antiphospholipid antibody syndrome. J. Autoimmun..

[23] Toubi E (2005). Livedo reticularis is a marker for predicting multi-system thrombosis in antiphospholipid syndrome. Clin. Exp. Rheumatol..

[24] Sneddon IB (1965). Cerebrovascular lesions and livedo reticularis. Br. J. Dermatol..

[25] Francès C (1999). Sneddon syndrome with or without antiphospholipid antibodies. A comparative study in 46 patients. Med. (Baltim.).

[26] Boortz-Marx RL, Clark HB, Taylor S, Wesa KM, Anderson DC (1995). Sneddon's syndrome with granulomatous leptomeningeal infiltration. Stroke.

[27] Hilton DA, Footitt D (2003). Neuropathological findings in Sneddon's syndrome. Neurology.

[28] Rutter-Locher Z (2016). Sneddon's syndrome: It is all in the ectoderm. Pract Neurol..

[29] Macário F (1997). Sneddon's syndrome: A vascular systemic disease with kidney involvement?. Nephron.

[30] Ruiz-Irastorza G (2011). Evidence-based recommendations for the prevention and long-term management of thrombosis in antiphospholipid antibody-positive patients: Report of a task force at the 13th International Congress on antiphospholipid antibodies. Lupus.

[31] Tumian NR, Hunt BJ (2022). Clinical management of thrombotic antiphospholipid syndrome. J. Clin. Med..

[32] Hughes GR, Cuadrado MJ, Khamashta MA, Sanna G (2001). Headache and memory loss: Rapid response to heparin in the antiphospholipid syndrome. Lupus.

[33] Hughes GR (2003). Migraine, memory loss, and "multiple sclerosis". Neurological features of the antiphospholipid (Hughes') syndrome. Postgrad. Med. J..

[34] Schofield JR, Hughes HN, Birlea M, Hassell KL (2021). A trial of antithrombotic therapy in patients with refractory migraine and antiphospholipid antibodies: A retrospective study of 75 patients. Lupus.

[35] Edwards MH (1997). Hydroxychloroquine reverses thrombogenic properties of antiphospholipid antibodies in mice. Circulation.

[36] Espinola RG, Pierangeli SS, Gharavi AE, Harris EN (2002). Hydroxychloroquine reverses platelet activation induced by human IgG antiphospholipid antibodies. Thromb. Haemost..

[37] Rand JH (2008). Hydroxychloroquine directly reduces the binding of antiphospholipid antibody-beta2-glycoprotein I complexes to phospholipid bilayers. Blood.

[38] Schmidt-Tanguy A (2013). Antithrombotic effects of hydroxychloroquine in primary antiphospholipid syndrome patients. J. Thromb. Haemost..

[39] Nuri E (2017). Long-term use of hydroxychloroquine reduces antiphospholipid antibodies levels in patients with primary antiphospholipid syndrome. Immunol. Res..

[40] Kravvariti E, Koutsogianni A, Samoli E, Sfikakis PP, Tektonidou MG (2020). The effect of hydroxychloroquine on thrombosis prevention and antiphospholipid antibody levels in primary antiphospholipid syndrome: A pilot open label randomized prospective study. Autoimmun. Rev..

[41] Broder A, Putterman C (2013). Hydroxychloroquine use is associated with lower odds of persistently positive antiphospholipid antibodies and/or lupus anticoagulant in systemic lupus erythematosus. J. Rheumatol..

[42] Andrade D, Tektonidou M (2016). Emerging therapies in antiphospholipid syndrome. Curr. Rheumatol. Rep..

[43] Ben-Zvi I, Kivity S, Langevitz P, Shoenfeld Y (2012). Hydroxychloroquine: From malaria to autoimmunity. Clin. Rev. Allergy Immunol..

[44] de Groot PG, Derksen RH (2005). Pathophysiology of the antiphospholipid syndrome. J. Thromb. Haemost..

[45] Tektonidou MG (2019). EULAR recommendations for the management of antiphospholipid syndrome in adults. Ann. Rheum. Dis..

[46] Furie KL (2011). Guidelines for the prevention of stroke in patients with stroke or transient ischemic attack: A guideline for healthcare professionals from the american heart association/american stroke association. Stroke.

[47] Saraiva-Sda S (2015). Recurrent thrombosis in antiphospholipid syndrome may be associated with cardiovascular risk factors and inflammatory response. Thromb. Res..

[48] Cervera R, Boffa MC, Khamashta MA, Hughes GR (2009). The Euro-Phospholipid project: Epidemiology of the antiphospholipid syndrome in Europe. Lupus.

