# Supplementary material for: Prevalence, risk factors, and prognosis of central nervous system manifestations in antiphospholipid syndrome
Source: Sci Rep. 2023 Jun 1;13:8915. doi: 10.1038/s41598-023-35955-2 (PMC10235119; doi:10.1038/s41598-023-35955-2)
Supplement: Supplementary file 1 — Supplementary Tables. [file 41598_2023_35955_MOESM1_ESM.doc]

**Prevalence, risk factors, and prognosis of central nervous system manifestations in antiphospholipid syndrome**

Meige Liu^1^, Gongming Li^2^, Xiaodong Song^1^, Yangyi Fan^1^ & Chun Li^3^

^1^ Department of Neurology, Peking University People’s Hospital, Beijing, China.

^2^ Department of Rheumatology and Immunology, Linyi Traditional Chinese Medicine Hospital, Shandong, China.

^3^ Department of Rheumatology and Immunology, Peking University People’s Hospital, Beijing, China.

**Supplementary Table 1.** The clinical manifestations of SLE patients in this study.

| Variable |  |
| --- | --- |
| Mucocutaneous symptoms, n (%) | 59 (46.8) |
| Arthritis, n (%) | 57 (45.2) |
| Hematological abnormalities, n (%) | 53 (42.1) |
| Fever, n (%) | 34 (27.0) |
| Neuropsychiatric manifestations, n (%) | 29 (23.0) |
| Lupus nephritis, n (%) | 18 (14.3) |
| Serositis, n (%) | 11 (8.7) |
| Mean dose of corticosteroids, (mg/d) | 38.6 |

*SLE s*ystemic lupus erythematosus.

**Supplementary Table 2.** The treatment of other autoimmune disease in this study.

| Treatments |  |
| --- | --- |
| Corticosteroids, n (%) | 28 (71.8) |
| Hydroxychloroquine, n (%) | 21 (53.8) |
| Cyclophosphamide, n (%) | 9 (23.1) |
| Mycophenolate mofetil, n (%) | 5 (12.8) |
| Intravenous gamma globulin, n (%) | 5 (12.8) |
| Cyclosporine, n (%) | 4 (10.3) |
| Leflunomide, n (%) | 4 (10.3) |
| Methotrexate, n (%) | 2 (5.1) |
| Azathioprine, n (%) | 2 (5.1) |

**Supplementary table 3.** Demographic and clinical features of APS patients with ischemic stroke

|  | Overall  (n=79) | recurrent stroke (n=10) | non-recurrent stroke (n=69) | *P value* |
| --- | --- | --- | --- | --- |
| Age at diagnosis (years) | 51.1 ± 15.9 | 44.5 ± 17.0 | 51.9 ± 15.6 | 0.108 |
| Male gender, n (%) | 23 (29.1) | 5 (50.0) | 18 (26.1) | 0.145 |
| Disease duration (months) | 12 (1, 48) | 46 (4.75, 75) | 10 (1, 48) | 0.506 |
| Secondary APS, n (%) | 50 (63.3) | 7 (70.0) | 43 (62.3) | 0.738 |
| Extracranial arterial events, n (%) | 35 (44.3) | 6 (60.0) | 29 (42.0) | 0.325 |
| Venous events, n (%) | 33 (41.8) | 5 (50.0) | 28 (40.6) | 0.734 |
| Obstetric events, n (%) | 17 (21.5) | 3 (30.0) | 14 (20.3) | 0.441 |
| Livedo reticularis, n (%) | 7 (8.9) | 3 (30.0) | 4 (5.8) | 0.040 |
| APS nephropathy, n (%) | 4 (5.1） | 2 (20.0) | 2 (2.9) | 0.076 |
| Thrombocytopenia, n (%) | 29 (36.7） | 1 (10.0) | 28 (40.6） | 0.083 |
| Diabetes, n (%) | 18 (22.8) | 2 (20.0) | 16 (23.2) | 1.000 |
| Hypertension, n (%) | 35 (44.3) | 4 (40.0) | 31 (44.9) | 1.000 |
| Dyslipidaemia, n (%) | 60 (75.9) | 9 (90.0) | 51 (73.9) | 0.437 |
| Smoking, n (%) | 15 (19.0) | 4 (40.0) | 11 (15.9) | 0.089 |
| Obesity, n (%) | 7 (8.9) | 1 (10.0) | 6 (8.7) | 1.000 |
| aβ2GPI, n (%) | 50 (63.3) | 8 (80.0) | 42 ( 60.9) | 0.310 |
| aCL, n (%) | 58 (73.4) | 6 (60.0) | 52 (75.4) | 0.443 |
| LA, n (%) | 59 (74.7) | 9 (90.0) | 50 (72.5) | 0.438 |
| Triple aPL positivity, n (%) | 30 (38.0) | 5 (50.0) | 25 (36.2) | 0.492 |
| Antiplatelet drugs, n (%) | 30 (38.0) | 5 (50.0) | 25 (36.2) | 0.492 |
| Anticoagulants, n (%) | 38 (48.1) | 5 (50.0) | 33 (47.8) | 1.000 |
| Corticosteroids, n (%) | 62 (78.5) | 8 (80.0) | 54 (78.3) | 1.000 |
| Hydroxychloroquine, n (%) | 40 (50.6) | 6 (60.0) | 34 (49.3) | 0.737 |
| Immunosuppressants, n (%) | 68 (86.1） | 9 (90.0) | 59 (85.5） | 1.000 |
| Statins, n (%) | 11 (13.9) | 2 (20.0) | 9 (13.0) | 0.624 |

Data were presented as the mean ± standard deviation, median (interquartile range) or n, where n = number of patients. *APS* antiphospholipid syndrome, *aβ2GPIs* anti-β2 glycoprotein I antibodies, *aCL* anticardiolipin antibody, *LA* lupus anticoagulant, *aPL* antiphospholipid antibody.
